# Moulting Performances Evaluation of Female Orange Mud Crab, *Scylla olivacea* (Herbst, 1796) In-Captivity: Effects of Water Salinity and Limb Autotomy

**DOI:** 10.21315/tlsr2024.35.1.11

**Published:** 2024-03-30

**Authors:** Amin-Safwan Adnan, Lawrencia Casey Gamburud, Izzah-Syafiah Mohd Affendi, Mardhiyyah Mohd Pauzi, Hairul Hafiz Mahsol, Taufik Muhammad, Hidayah Manan, Muhammad Naimullah, Che-Zulkifli Che Ismail, Muhd-Farouk Harman, Muhammad Nur Syafaat, Nadirah Musa, Hongyu Ma, Mhd Ikhwanuddin

**Affiliations:** 1 Department of Applied Sciences and Agriculture, Tunku Abdul Rahman University of Management and Technology, Johor Branch, Jalan Segamat/Labis, 85000 Segamat, Johor, Malaysia; 2 Faculty of Fisheries and Food Sciences, Universiti Malaysia Terengganu, 21030 Kuala Nerus, Terengganu, Malaysia; 3 Higher Institution Centre of Excellence (HICoE), Institute of Tropical Aquaculture and Fisheries, Universiti Malaysia Terengganu, 21030 Kuala Nerus, Terengganu, Malaysia; 4 Conservation Biology Program, Faculty of Tropical Forest, Universiti Malaysia Sabah, Jalan UMS, 88400 Kota Kinabalu, Sabah, Malaysia; 5 Department of Environmental Biology Fisheries Science, National Taiwan Ocean University, 2 Pei-Ning Rd., Keelung 20224, Taiwan, Republic of China; 6 Crustacean Aquaculture Research Division, FRI Pulau Sayak, Kota Kuala Muda, Kedah, Malaysia; 7 Impact Assessment Research Division, Fisheries Research Institute Batu Maung, Department of Fisheries, Pulau Pinang, Malaysia; 8 Research Institute for Brackishwater Aquaculture and Fisheries Extension (RIBAFE), Maros, 90512, South Sulawesi, Indonesia; 9 Guangdong Provincial Key Laboratory of Marine Biotechnology, Shantou University, Shantou 515063, China; 10 STU-UMT Joint Shellfish Research Laboratory, Shantou University, Shantou 515063, China

**Keywords:** Aquaculture, Crab Fattening, Limb Autotomy, Salinity, Soft-Shell Crabs, Akukultur, Penggemukan Ketam, Autotomi Badan, Kemasinan, Ketam Kulit Lembut

## Abstract

Female *Scylla olivacea* has become more popular in Malaysia as emerging species mainly for soft-shell crabs and crab fattening (to increase weight, size and ovary maturation so that they can be sold at a higher price). To harvest crabs in soft-shell conditions and fattening, both conditions depend mostly on moulting events. To accelerate

the moulting process, the manipulation of water parameter (salinity) and autotomy of the limb is commonly used. In this study, the evaluation of the moulting performances of full limb autotomy (the removal of all the appendages except for the swimming legs) and non-ablated (control) using immature *S. olivacea* cultured in three different salinity treatments (10 ppt, 20 ppt and 30 ppt) were performed. Results indicate there were significant differences between mud crab’s culture duration, BW increments, growth performances and feeding efficiency with salinity. However, CW increments and survival indicate no significant effect with salinity. Meanwhile, limb autotomy proved to affect the culture duration, BW increments, survival and feeding efficiency of *S. olivacea*. The study concludes that both salinity and limb autotomy play significant roles in moulting performances of *S. olivacea*, with 20 ppt being the best salinity to stimulate *S. olivacea* moulting and development compared with the other two treatments (10 ppt and 30 ppt). Limb autotomy also indicates promising results as this technique proved to accelerate the moulting duration of *S. olivacea* with a 100% moulting percentage within 30 days. Therefore, the outcome would certainly benefit in the aquaculture production of this species of commercial importance mainly on soft-shell crabs production and also emerge as crabs fattening technique.

## Abstract (ms)

*Scylla olivacea* betina telah menjadi lebih popular di Malaysia sebagai spesies yang baru muncul terutamanya untuk ketam kulit lembut dan penggemukan ketam (untuk meningkatkan berat, saiz dan kematangan ovari supaya ia boleh dijual pada harga yang lebih tinggi). Untuk menuai ketam dalam keadaan kulit lembut dan menggemukkan, kedua-dua keadaan bergantung kebanyakannya pada proses penyalinan kulit. Untuk mempercepatkan proses penyalinan kulit, manipulasi parameter air (kemasinan) dan autotomi anggota badan biasanya digunakan. Dalam kajian ini, penilaian prestasi penyalinan kulit autotomi anggota badan penuh (penyingkiran semua pelengkap kecuali kaki berenang) dan tidak ablat (kawalan) menggunakan *S. olivacea* tidak matang yang dikultur dalam tiga rawatan kemasinan yang berbeza (10 ppt, 20 ppt and 30 ppt) telah dilakukan. Keputusan menunjukkan terdapat perbezaan yang ketara antara tempoh kultur ketam lumpur, kenaikan BW, prestasi pertumbuhan dan kecekapan pemakanan dengan kemasinan. Walau bagaimanapun, kenaikan CW dan kemandirian menunjukkan tiada kesan ketara dengan kemasinan. Sementara itu, autotomi anggota badan terbukti mempengaruhi tempoh kultur, kenaikan BW, kemandirian dan kecekapan pemakanan *S. olivacea*. Kajian menyimpulkan bahawa kedua-dua kemasinan dan autotomi anggota badan memainkan peranan penting dalam prestasi penyalinan kulit *S. olivacea*, dengan 20 ppt merupakan kemasinan terbaik untuk merangsang *S. olivacea* moulting dan perkembangan berbanding dengan dua rawatan lain (10 ppt dan 30 ppt). Autotomi anggota badan juga menunjukkan hasil yang menjanjikan kerana teknik ini terbukti mempercepatkan tempoh penyalinan kulit *S. olivacea* dengan peratusan penyalinan kulit 100% dalam masa 30 hari. Oleh itu, hasilnya pasti akan memberi manfaat dalam pengeluaran akuakultur spesies berkepentingan komersial ini terutamanya dalam pengeluaran ketam kulit lembut dan juga muncul sebagai teknik penggemukan ketam.

**Highlights**

- Moulting performances of *S. olivacea* can be induced by both salinity and limb autotomy.
- We present the results on combination of both salinity and limb autotomy focusing on moulting duration and percentage, body weight and carapace width increments, growth performances and regeneration (physical completeness), survival and feeding efficiency of *S. olivacea*.
- This may serve as essential guideline for prospective mud crab hatchery production, especially on soft-shell crab production and crab fattening, as one of the main aquaculture industries for present and future prospect.

## INTRODUCTION

Mud crab genus Scylla (Brachyura: Portunidae) is well known as an economically important crustacean species that are fast-growing that attain larger size among portunids and are widely distributed through the Indo-Pacific region (Keenan *et al*. 1998; Ikhwanuddin *et al*. 2011). They inhabit intertidal mangrove forests with fluctuating salinity and support the livelihood of local fishery communities (Alberts-Hubatsch *et al*. 2016; Amin-Safwan *et al*. 2019a). Global capture production of Scylla is reported above 20,000 tonnes in the last decade (Food and Agriculture Organization of the United Nations [FAO] 2018), however, the global aquaculture production recorded just below 15,000 tonnes from 1980 until 2003. However, starting from 2004, the production increased exponentially to above 100,000 tonnes (Fishery Bureau of Ministry of Agriculture China 2012) and has been increasing steadily ever since (FAO 2018). Due to higher demand from the global market, it is expected that the growth of the Scylla aquaculture sector will continue to increase (Waiho et al. 2018). Orange mud crab, *S. olivacea*, is among one of the four species in genus Scylla and commercially important mud crab species especially in Southeast Asia such as Malaysia (Ikhwanuddin *et al*. 2011; Amin-Safwan *et al*. 2019b; Muhd-Farouk *et al*. 2019). Their habitat is the mangrove forest that has commonly been exposed to tidal changes (Keenan *et al*. 1998; Keenan 1999), with several studies reporting that this species commonly prefers lower-to-intermediate salinity (La Sara 2001; Amin-Safwan *et al*. 2019b).

Salinity is considered to be one of the most important abiotic factors in aquaculture, as most cultured crustacean species reported having some degree of euryhalinity (species-specific). Several studies explored the effect of salinity on the physiological regulation of mud crabs, their larval dispersal and recruitment, geographical distribution, behaviour, mating, reproduction, spawning and survival (Romano & Zeng 2006; Talpur & Ikhwanuddin 2012; Baylon 2013; Ikhwanuddin *et al*. 2014). Moreover, several studies also reported that salinity influenced the food intake efficiency and conservation activity (Mia & Shah 2010), fatty acids composition (Wu *et al*. 2019), ovarian maturation (Amin-Safwan *et al*. 2016; 2019b), immunity (Ma *et al*. 2013), and even parasitism infestation (Fazhan *et al*. 2017). Yet, there are only a few studies have evaluated the optimal salinity for moulting of mud crab (Jantrarotai *et al*. 2002; Gong *et al*. 2015), with most studies perform mainly focused on larval stages compared to juveniles and broodstock.

Several techniques were reported to enhance crab growth and development by inducing the crab to moult rapidly, such as the application of limb autotomy (Fujaya *et al*. 2020; Rahman *et al*. 2020), eyestalk ablation (Siti 2012), injection of plants extraction (Aslamyah & Fujaya 2010; Fujaya 2011), and involvement of hormones (Muhd-Farouk *et al*. 2019). Some chose to use preferable and painless way to increase the moulting process by manipulating the water parameter (salinity) and the amount of heavy metals in the water (Vernberg *et al*. 1973; Madsen & Shine 1992; McGee *et al*. 1998; Gaude & Anderson 2011; Gong *et al*. 2015). However, Siti (2012) indicated that most of the aquaculturists, practitioners and crab breeders prefer to use the easy, budget-friendly and quickest way such as limb autotomy as this technique is proven to induce crabs to moult within three to four weeks. Up to date, there are only several studies conducted on limb autotomy of *S. olivacea* such as by Fujaya *et al*. (2020) and Rahman *et al*. (2020), however, no further information regarding the effect on moulting performances using limb autotomy associated with salinity was reported.

Therefore, the present study was designed mainly to evaluate the effects of water salinity and limb autotomy on moulting performances of *S. olivacea*. The moulting percentage and duration, increment sizes consist of body weight and carapace width, growth performances and physical completeness after moulting, survival rate and feeding efficiency were identified and evaluated. The results gained may support the development and improvement of soft-shell productions and as one of the fattening techniques, besides for maintenance and technology development focusing on mud crab (*S. olivacea*) growth in-captivity based on the optimal salinity and autotomy practice. The outcome of this study would certainly benefit the aquaculture production of this species of commercial importance.

## MATERIALS AND METHODS

### Mud Crab Samples

A total of 360 immature females *S. olivacea* (intermoult phase; carapace width, CW < 9.0 cm; body weight, BW 80 g–100 g) were sampled from Setiu Wetlands Mangrove Forest, Terengganu, Malaysia (5°40′47.93″N 102°42′45.04″E). *S. olivacea* identification was based on the characteristics and morphological description by Keenan *et al*. (1998). The selected experimental immature CW was based on the size at maturity of *S. olivacea* (8.4 cm–9.2 cm based on gonadal maturation), which was determined according to previous studies by Ikhwanuddin *et al*. (2010) and Waiho *et al*. (2016) from the same sampling site. The maturity status was assessed according to the abdomen shape and colouration (Amin-Safwan *et al*. 2019b). The selected CW and BW of the experimental crabs used in the present study confirmed those of crabs used by commercial farmers for soft-shell crab production and crab fattening in Malaysia (personal communication).

The selection of intermoult crabs was based on the moult stage classification of *S. serrata* by Quinitio and Estepa (2011). Intermoult crabs (C_3_) exhibit a completely hardened exoskeleton and minimal epidermal separation from the swimming cuticle (Fujaya *et al*. 2020). Crabs that are in postmoult, early intermoult (C_1_–C_2_), late intermoult (C_4_), and premoult were not selected in this study, as McCarthy and Skinner (1977) reported that limb autotomy during the intermoult stage hastens the moulting process. The selected crabs were transferred to the Marine Hatchery of the Institute of Tropical Aquaculture and Fisheries (AKUATROP), Universiti Malaysia Terengganu, for subsequent analysis. The size of each crab was measured and recorded; external CW (the distance between the tips of the 9th anterolateral spine of the crab carapace) using a six-inch liquid crystal display (LCD) digital Vernier calliper (accuracy: 0.01 cm; Kingsmart brand, Hong Kong), whereas the BW was measured using a digital balance (accuracy: 0.01 g; Shimadzu model, Japan). Each crab was labelled with a cable tie tag (Nylon brand: 3 mm width × 150 mm length) on its swimming legs.

Setiu Wetlands is an open and free fishing area; thus, no licensing was required for the acquisition of the mud crabs (Waiho *et al*. 2017). We adhered to the Association for the Study of Animal Behaviour (ASAB) (2012) “Guidelines for the treatment of animals in behavioural research and teaching” published in “Animal Behaviour”. None of the work involved endangered or protected species. *S. olivacea* is a commercially available species of mud crab, so no special authorisation was required to acquire it.

### Salinity Acclimatisation and Treatments

There were three different salinity treatments (10 ppt, 20 ppt and 30 ppt), with each treatment consisting of three replicates (*n* = 20) of autotomised (both claws and all walking legs) and non-ablated *S. olivacea* (as control). Each crab was placed individually in an enclosed rectangular plastic container (36 cm length × 22 cm width × 21 cm height) to avoid cannibalism, kept in a large fibreglass tank (320 cm length × 138 cm width × 60 cm height) with a recirculating water system. The moulting duration, moulting percentage, CW and BW increments, growth performances and regeneration of limbs, survival, and feeding efficiency was measured and recorded for each treatment.

Crabs were first acclimated to lower salinity accordingly following method Amin-Safwan *et al*. (2019b) before each treatment. For Treatment 1 (10 ppt), the crabs were acclimated at 30 ppt for five days and continued at lower salinities of 25 ppt, 20 ppt, 15 ppt and 10 ppt for five days each before being transferred into the 10 ppt treatment. As for Treatment 2 (20 ppt), the crabs were acclimated at 30 ppt, 25 ppt and 20 ppt for five days each before being introduced to the 20 ppt treatment, and for Treatment 3 (30 ppt), the crabs were acclimated at 30 ppt for five days before starting day 1 of the treatment.

Seawater was filtered and diluted with filtered freshwater until the appropriate salinity level for each treatment was achieved (10 ppt, 20 ppt and 30 ppt), continued with chlorine treatment (overnight), then dechlorinated using anti-chlorine. The treated seawater was then vigorously aerated overnight. Water parameters, such as temperature, dissolved oxygen (DO), pH, conductivity, oxidation-reduction potential (ORP), and salinity were determined and monitored daily using a YSI 556 MPS multi-probe meter (YSI Incorporated, Ohio), and a refractometer (ATAGO, Japan).

The crabs (autotomised and non-ablated) were cultured under treatment with different salinities (10 ppt, 20 ppt and 30 ppt), in ambient temperature (27°C–29°C), and in dark photoperiod (tanks were fully covered with black nylon netting except for feeding times) in the hatchery. Water exchange was done 100% once a week, as the use of a recirculating water system was applied. The crabs were fed with chopped Yellow stripe scad fish, *Selaroides leptolepis*, at 10% body weight twice daily (0800 h and 1800 h), and the uneaten food was collected and weighed for feeding efficiency determination. Crabs monitoring were done four times a day (morning – 0800 h; afternoon – 1300 h; evening – 1800 h; midnight – 0000 h).

### Limb Autotomy Technique

**Figure 1:**
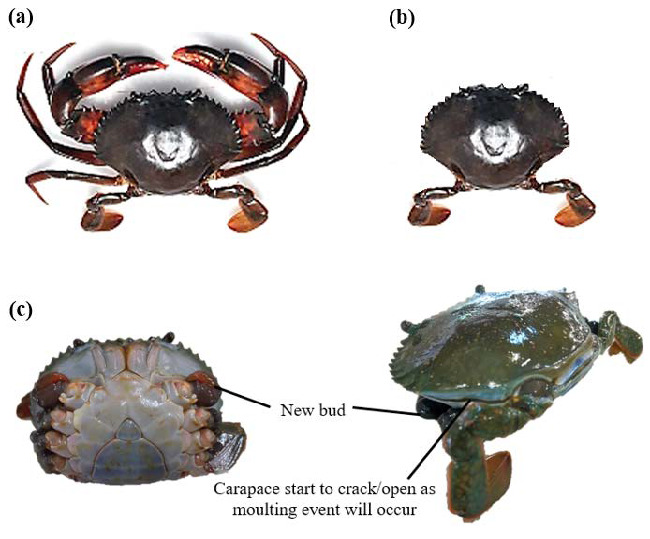
(a) Non-ablated *S. olivacea*, (b) autotomised *S. olivacea* (both claws/chelipeds and all walking legs were removed, leaving only the pleopods), and (c) the new limb bud growth and carapace cracking event as moulting period neared for the autotomised crabs.

During the limb autotomy technique, the chelipeds (claws) and pereiopods (walking legs) of 180 immature crabs were removed by applying the ablation, leaving only the swimming legs, mainly for their movement (Fig. 1). This type of ablation (both claws and all walking legs) was chosen for its efficiency instead of other ablation techniques (unilateral claw, unilateral walking legs, bilateral claws and bilateral walking legs) as reported by Rahman *et al*. (2020).

### Estimation of the Growth Performances

Over the experiment, the number of dead crabs and the culture duration was observed and recorded every day, and the crabs were weighed after their body was completely hardened (approximately ± 7 days) to reduce stress. The experiment was terminated once all of the crabs successfully moulted; since all of the autotomised crabs moulting duration were obtained less than 30 days, therefore the moulting duration of 30 days was used as a control period for the non-ablated crabs (only crabs moulted within 30 days were recorded for the non-ablated crabs). The culture duration (days), moulting percentage (%), the increments percentage of CW (%) and BW (%), specific growth rate (SGR), survival rate (%), and feeding efficiency (FE) were calculated based on the following formulas (Rahman *et al*. 2020):


$$\begin{array}{lll} \text{Culture duration} & = & {\text{D}}_{2}-{\text{D}}_{\text{1}}\\ \text{Moulting percentage} & = & 100\times (\text{no.}\;\text{of moulted crab}/\text{total no}\text{. of crab})\\ \text{CW increment }(\%) & = & 100\times \;[({\text{CW}}_{\text{n}}-{\text{CW}}_{\text{n-}1})/({\text{CW}}_{\text{n-}1})]\\ \text{BW increment }(\%) & = & 100\times [({\text{BW}}_{\text{n}}-{\text{BW}}_{\text{n-}1})/({\text{BW}}_{\text{n-}1})]\\ \text{SGR }(\%\;{\text{day}}^{-1}) & = & 100\times ({\text{BW}}_{\text{n}}-{\text{BW}}_{\text{n-}1})/\text{t}\\ \text{Survival rate }(\%) & = & 100\times (\text{final no}\text{. of crab}/\text{initial no}\text{. of crab})\\ \text{FE }(\%) & & 100\times ({\text{BW}}_{\text{n}}-{\text{BW}}_{\text{n-}1})/\text{C} \end{array}$$

Where D_1_ is the date of treatments started on crabs, D_2_ is the date of crabs successfully moulted, BW_n_ is the body weight (g) after moulting, BW_n-1_ is the body weight (g) before moulting, CW_n_ is the carapace width (cm) after moulting, CW_n-1_ is the carapace width (cm) before moulting, t is the length of culture duration (days), and C is the amount of food intake (fish) (g) in a whole culture period (Rahman *et al*. 2020).

### Data Analysis

All data analysis was conducted using the Statistical Package for Social Sciences (SPSS) for Windows, version 25.0 (IBM, Armonk, NY, USA). Data were presented as average ± standard deviation. All data were tested for the normality of distribution and homogeneity of variance using the Shapiro-Wilk and Kolmogorov- Smirnov Test, respectively. One-Way Analysis of Variance (ANOVA) was conducted for normal distribution data, followed by Tukey’s multiple range test to determine the differences between each treatment. When normal distribution and/ or homogeneity of variances were not achieved, the data were subjected to the Kruskal-Wallis nonparametric test, followed by the Pairwise multiple comparison test. The Mann-Whitney U test was used to access the differences between non-ablated and limb autotomy treatments. The Chi-Square Test was used to identify the survival of moulted crabs in different salinity treatments. A value of *p* < 0.05 was considered statistically significant.

## RESULTS

### The Length of Culture Duration and Moulting Percentage

Results indicate the culture duration of ablated crabs in each salinity treatment were less than 30 days with each treatment managing to produce a 100% moulting percentage. As for the non-ablated crabs, due to the result obtained by the ablated crabs (< 30 days), therefore only the moulted crabs within 30 days duration were counted and recorded, with 20.00 ± 5.00%, 36.67 ± 5.70%, and 13.33 ± 2.87% moulting percentage for 10 ppt, 20 ppt and 30 ppt respectively. There were significant differences between culture duration of the ablated crabs (ANOVA: *p* = 0.029; *p* < 0.05) and non-ablated crabs (ANOVA: *p* = 0.000; *p* < 0.05) with salinity treatments. Overall, statistical analysis indicates that there were significant differences between culture duration and salinity treatments (Kruskal-Wallis Test: *p* = 0.000; *p* < 0.05), and between non-ablated and ablated treatment (Mann-Whitney U Test: *p* = 0.000; *p* < 0.05) (Table 1).

### The Carapace Width and Body Weight Increments

As for CW increments (Table 2), result obtained shows there were no significant differences between ablated crabs (ANOVA: *p* = 0.916; *p* > 0.05) and non-ablated crabs (ANOVA: *p* = 0.177; *p* > 0.05) with salinity treatments. Overall results also showed that there were no significant differences (Kruskal-Wallis Test: *p* = 0.226; *p* > 0.05) between CW and salinity treatments, and between non-ablated and ablated treatment (Mann-Whitney U Test: *p* = 0.940; *p* > 0.05). For the non-ablated crabs, 10 ppt recorded the highest percentage of CW increments, followed by 20 ppt, and lastly 30 ppt. However, for the ablated crabs, different results were obtained, as 20 ppt recorded as the highest increment percentage, followed by 10 ppt, and lastly 30 ppt.

There was significant difference between BW increments of the ablated crabs with salinity treatments (ANOVA: *p* = 0.011; *p* < 0.05), however, no significant result recorded for non-ablated crabs (ANOVA: *p* = 0.547; *p* > 0.05). However, overall results recorded significant differences (Kruskal-Wallis Test: *p* = 0.005; *p* < 0.05) between BW and salinity treatments, and between non-ablated and ablated treatment (Mann-Whitney U Test: *p* = 0.015; *p* < 0.05). For the non-ablated crabs, 20 ppt recorded the highest percentage of BW increment, followed by 30 ppt, and lastly 10 ppt. However, for the ablated crabs, different results were obtained as 30 ppt recorded the highest increment percentage, followed by 20 ppt and 10 ppt (Table 3).

### The Growth Rate and Regeneration of Limbs

Results of specific growth rate (SGR) indicated 20 ppt as the best salinity treatment both for non-ablated and ablated crabs, followed by 30 ppt, and the least was 10 ppt treatment (Table 4). There were no significant differences of SGR for both of the non-ablated crabs (ANOVA: *p* = 0.153; *p* > 0.05) and ablated crabs (ANOVA: *p* = 0.051; *p* > 0.05) with salinity treatments. No significant difference was also recorded between non-ablated and ablated treatment (Mann-Whitney U Test: *p* = 0.937; *p* > 0.05). However, overall analysis indicates a significant difference between SGR and salinity treatments (Kruskal-Wallis Test: *p* = 0.007; *p* < 0.05).

**Figure 2:**
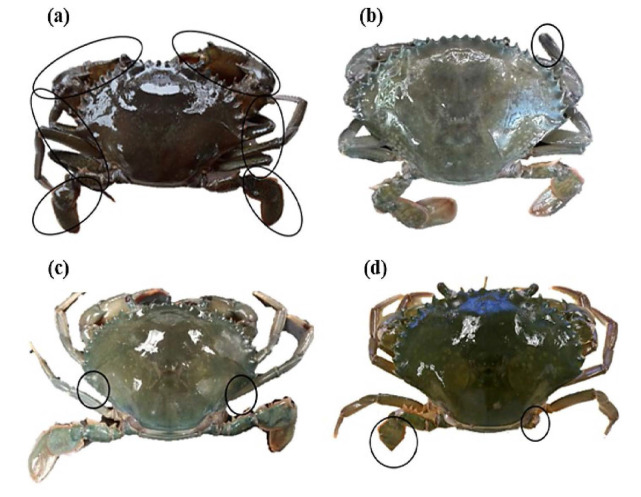
Regeneration of limbs of moulted female *S. olivacea* without ablation and with the induction of limb autotomy practice, (a) Completed growth (consist of a pair of chelipeds, three pairs of pereiopods and a pair of pleopods), (b–d) uncompleted growth (missing of some parts of pereiopods and pleopods). Circles in (a) indicates the complete of regeneration of body parts of mud crab, meanwhile the circles of (b) indicates the incomplete regeneration of a walking leg, circles in (c) indicates the incomplete regeneration of a pair of walking legs, and circles in (d) indicates the incomplete regeneration of pleopods of *S. olivacea* found in the present study.

As for the regeneration of limbs, the moulting of non-ablated crabs indicated 100% regeneration of claws and walking legs. Meanwhile, for the ablated crabs, results indicate that only 85% of crabs managed to regenerate their claws and walking legs successfully, with the remaining (15%) failing to regenerate completely (uncomplete physical appearances: without complete numbers of claws, walking legs, and/or swimming paddles) (Fig. 2). It was observed that the regenerated appendages for non-ablated crabs were similar (100%) to their original size, however, different results were obtained for the ablated crabs as most of the moulted crabs (95%) shows relatively smaller claws and walking legs compared to the non-ablated moulted crabs.

### Survival

The effects of water salinity and limb autotomy on the survival of *S. olivacea* were presented in Table 5. There was significant difference between survival of non-ablated and ablated crabs (Chi-square test: χ^2^ = 18.947, df = 1, *p* < 0.01). Compared to the survival of non-ablated crabs in different salinity treatments; each treatment recorded 100% survival of *S. olivacea* (10 ppt, 20 ppt and 30 ppt), however, the survival of ablated crabs in different salinity treatments recorded different results, with 10 ppt recorded as the highest survival, followed by 20 ppt, and lastly 30 ppt. However, there was no significant difference recorded between the survival of ablated crabs with salinity treatments (Chi-square test: χ^2^ = 4.815, df = 2, *p* > 0.05). As for non-ablated crabs, no Chi-square (χ^2^) value was obtained as this treatment recorded 100% survival, thus resulting in constant value and no pattern was observed. Overall, there was no significant difference (Chi-square test: χ^2^ = 4.561, df = 2, *p* > 0.05) recorded between survival and salinity treatments.

### Feeding Efficiency

For the non-ablated crabs, 20 ppt recorded the highest feeding efficiency, followed by 30 ppt and lastly 10 ppt. However, for the ablated crabs, different results were obtained as the highest feeding efficiency was recorded in 30 ppt, followed by 20 ppt, and lastly 10 ppt (Table 6). Both treatments (feeding efficiencies of non-ablated crabs and ablated crabs) indicated no significant differences with salinity treatments (Kruskal-Wallis Test: *p* = 0.059; *p* > 0.05, and *p* = 0.056; *p* > 0.05, respectively). However, the overall analysis reported that there were significant differences between feeding efficiency with salinity treatments (Kruskal-Wallis Test: *p* = 0.006; *p* < 0.05), and between non-ablated and ablated treatment (Mann-Whitney U Test: *p* = 0.029; *p* < 0.05).

## DISCUSSION

Mud crabs grow by shedding off their old exoskeleton and replacing it with a new one, through a periodic period known as the moulting cycle (Chang & Mykles 2011; Rahman *et al*. 2020). Previous studies reported that optimal salinity (Parado-Estepa & Quinitio 2011; Gong *et al*. 2015) and appendages ablation (He *et al*. 2016; Rahman *et al*. 2020) does influence faster and frequent moulting in mud crabs. As crab needs to regenerate limbs quickly, they will store part of nutrition which therefore causes a shortened moulting period (He *et al*. 2016), and with the aid of optimum salinity practice, the absorption of important nutrition and water’s nutrient into the crab’s body might be possible at a faster rate. The results of the present study clearly showed that ablation and salinity treatments significantly shorten the moulting duration and influence the moulting percentage of *S. olivacea*. Comparably, autotomised crabs indicated better results of moulting duration (faster and less than 30 days) with 100% moulting percentage for each salinity treatment, meanwhile, the non-ablated crabs recorded 20.00 ± 5.00% (10 ppt), 36.67 ± 5.70% (20 ppt), and 13.33 ± 2.89% (30 ppt) moulting percentage within 30 days treatment.

Based on a previous study done by Romano and Zeng (2006), higher salinities show a prolonged moulting period compared to lower salinities, same as recorded in the present study, as the highest salinity treatment (30 ppt) recorded the longest moulting period with an average of 27.28 ± 4.08 days (autotomised crabs) and 28.88 ± 1.36 days (non-ablated crabs). Dissimilarity to study done upon blue crab, *Callinectes sapidus* reported that the moulting duration showed a significantly shorter period at higher salinity (Chazaro-Olvera & Peterson 2004). Romano and Zeng (2006) reported that at 20 ppt, the moulting period of portunid crabs (*Portunus pelagicus*) for growth, development, and survival have an immediate and significant effect which was similar with the present study, where 20 ppt (both autotomised and non-ablated crabs) was the shortest moulting period recorded for each category treatment. However, 20 ppt of autotomised crabs (23.55 ± 2.92 days) recorded a faster moulting duration compared to the 20 ppt of non-ablated crabs (27.18 ± 1.62 days). The obtained results were consistent with previous findings on ablated crustaceans such as on *Eriocheir sinensis*, *Panulirus longipes*, *Jasus lalandii*, and *P. pelagicus* (Quinitio & Estepa 2011; Nadiah *et al*. 2012; He *et al*. 2016), as ablated individuals highly produced better moulting duration compared to the non-ablated. Generally, mud crabs store energy from daily feeding to increase their body size during moulting (He *et al*. 2013). To generate new appendage quickly, mud crab could embark on moulting when just part of nutrition is stored. This statement was supported by He *et al*. (2016) on *E. sinensis* juvenile, as only part of nutrition restored does enough to initiate and influenced them to moult, thus signifying the shortened moulting period. In addition, claw ablation and appendage ablation are reported as an effective way to reduce the moulting duration both in freshwater crab, *Oziothelphusa senex senex* (Hosamani *et al*. 2016), and the same species of *S. olivacea* (Rahman *et al*. 2020). However, Gong *et al*. (2015) reported a contradictory result, as appendages ablation prolonged the moulting duration of juvenile *S. paramamosain*.

As for the moulting percentage, all of the autotomised crabs in different salinity treatments (10 ppt, 20 ppt and 30 ppt) managed to produce 100% moulting success (less than 30 days), however, different results were obtained for the non-ablated crabs as 20 ppt recorded as the highest moulting percentage with 36.67 ± 5.7 %, followed by 10 ppt (20 ± 5%), and the least was 30 ppt (13.33 ± 2.87%). The dissimilarity might be due to the autotomised crabs crucially focused on obtained energy mainly for the limb growth development, meanwhile, the non-ablated crabs might shift the energy obtained for other purposes such as ovarian development rather than for moulting, thus signifying the roles of autotomy as catalyst and inducer for moult event. Hopkins (1982) also does agree, as he reported that the losses of important and large chelipeds as an example for mud crabs, genus *Scylla* has hastened the onset of the next moult. However, the effect of salinity and appendage autotomy on moult interval might depend not only on the individual maturation stage, moult stage (Quinitio & Estepa 2011), the number of appendages and types of appendage lost (Bennett 1973), and the age of the crab (Mariappan *et al*. 2000); as these physiological differences might different for species, or more specifically different from one individual to another.

A study upon salinity impact on the growth of *S. serrata* showed that higher water salinity has significantly increased BW compared to a lower water salinity (Mia & Shah 2010). Similar findings were obtained in the present study as the intermediate salinity (20 ppt) was recorded as the highest average of BW on the non-ablated crab (9.56 ± 2.46 g), meanwhile 30 ppt for the autotomised crabs (10.57 ± 8.36 g). Hopkins (1982) reported that autotomy of all crab appendages except swimming legs significantly reduced crab’s BW increment after moulting, although this may be even up by having shorter moulting intervals; similar results were obtained in both 10 ppt and 20 ppt. However, interestingly, a contradictory result was obtained in 30 ppt of autotomised crabs, as this treatment recorded the highest increment of BW among other treatments. This might correlate with the feeding efficiency as 30 ppt recorded the highest for the autotomy crabs, which signifying the levels of nutrient obtained. A study by Quinitio and Estepa (2011) also showed that autotomised *S. serrata* juveniles experienced lower specific growth rates after first and second moult in comparison with intact juveniles. Mykles (2001) added that intact females moulted faster than intact males and this may be attributed to the requirement for the female crabs to moult and hasten maturity to copulate and spawn. From present findings, limb autotomy might reduce the moulting intervals but at the cost of smaller chelipeds size, smaller carapace size, and lighter body weight. A previous study was done by Romano and Zeng (2006) on *P. pelagicus* showed that average CW was not significant at higher and lower salinities, however, at intermediate salinity, there was a significant difference. However, on contrary, there were no significant results recorded for CW increment of *S. olivacea* in the present study.

The salinity and autotomy did affect the feeding efficiency of *S. olivacea* in the present study. However, the feeding efficiency recorded contradict expectation; as autotomy would result in increased feeding response (reflecting the need for increased energy stores to facilitate regeneration). However, compared to the non-ablated crabs (highest in 20 ppt; 40.98 ± 10.38 %/day), autotomised crabs recorded slight lesser feeding efficiency in both 10 ppt and 20 ppt, with the highest recorded in 30 ppt (42.52 ± 3.96 %/day). A similar finding was reported by Darnell *et al*. (2018) on fiddler crab, *Leptuca pugilator*, as they observed feeding was greatly reduced for at least 96 h following autotomy of the major claw. However, Darnell *et al*. (2018) stressed the possibility of just a temporary effect on the feeding inhibition recorded. Moreover, Lemos *et al*. (2001) and Sang and Fotedar (2004) have demonstrated reduced growth at high salinities to appetite and reduced food assimilation respectively on prawns, *Farfantepenaeus paulensis* and *Penaeus latisulcatus*, respectively. Salinity proven affected food intake and conversion efficiency (Amin-Safwan *et al*. 2019b). Added with the appendages loss, the salinity treatments might reflect on the physiological of crabs, thus affecting the motivation to feed (Darnell *et al*. 2018).

Salinity is known as an imperative ecological factor for estuarine crabs as it might impact growth development through its effect on food intake, conversion efficiency and activity (Amin-Safwan *et al*. 2019b). Moreover, it was observed that salinity does affect the growth rate of *S. olivacea* in the present study. Comparably, the autotomised crabs showed a higher growth rate than non-ablated crabs, with 20 ppt being the best among other treatments (10 ppt and 30 ppt) in both conditions. Such improvement in autotomised crabs might be related to energy reservation due to less aggressiveness of crabs and a small amount of activities (limited due to ablated parts), this resulted in less consumption of energy which indirectly converted the main nutrition into body growth (He *et al*. 2016; Rahman *et al*. 2020). Similar findings were also obtained by Rahman *et al*. (2020), as their study indicated that SGR was significantly higher in all appendages ablated compared to parts of ablated (only claws or walking legs) and non-ablated crabs. Moreover, He *et al*. (2016) also reported the positive correlation between SGR with the number of autotomised limbs, indicating that more parts of autotomized limbs resulted in higher SGR production.

Regeneration is a remarkable adaptation of crustaceans to replace damaged limbs (Quinitio & Estepa 2011). Regeneration following autotomy in crustaceans has been well described in works of literature, basically consisting of basal growth, wound healing and proecdysial growth (Hopkins 1982; Skinner 1985; Quinitio & Estepa 2011), and that implies the same for *S. olivacea*. The development of the limb bud is the initial response to autotomy, which continue to grow until successful moult. Moreover, moulting performs other functions besides growth, such as the repair of damaged appendages, removal of metabolites, and preparation for incubation (Hartnoll 1982). In the present study, all autotomised crabs in the different salinity treatments managed to regenerate their appendages after moulting. However, comparably to the non-ablated crabs, most of the newly moulted crabs from autotomy produced smaller sizes of chelipeds (claws) and walking legs (95%), meanwhile, the non-ablated crabs produced the normal sizes of regeneration (100%). According to He *et al*. (2016), the regenerated claw of mud crab is oftentimes smaller after the moult immediately following the limb loss, and become normal after the second moult. The limb bud unfolds and expands, however the size of the limb bud is normally slightly smaller than the normal limb (Quinitio & Estepa 2011). Previous studies also reported that some factors influence the regeneration of lost limbs to their original size, such as the age or size of the individual (Mariappan *et al*. 2000), the number of limbs lost (Fingerman & Fingerman 1974; Amin-Safwan *et al*. 2019a), and the moult stage (Hopkins 1982).

The survivability of an organism highly depends on its acclimation and adaptation ability toward surroundings, and salinity does react as one of the important parameters for the aquatic living organism (Amin-Safwan *et al*. 2016; 2019b). Previous studies reported that salinity (Parado-Estepa & Quinitio 2011; Chand *et al*. 2015) and limb autotomy (Fujaya *et al*. 2020; Rahman *et al*. 2020) generally affected the survival of crustaceans by leading to low immunity and increased vulnerability to pathogenic bacteria (Juanes & Smith 1995; Slos *et al*. 2008). However, it is believed that risks will only be occurred outside the tolerance ranges of species, as each species has its own tolerances-specific. During the present study, it was not observed high mortality rate in all treatments; 100% survival rate for the non-ablated crabs in each salinity treatment (10 ppt, 20 ppt and 30 ppt), meanwhile, 95.00 ± 8.66% (10 ppt), 91.67 ± 14.43% (20 ppt) and 83.33 ± 14.43% (30 ppt) for the autotomised crabs. Each crab was stocked individually and provided with sufficient feed, with the water quality were well maintained. The present study, however, recorded no significant difference between salinity and survival of moulted *S. olivacea*. The majority of mortalities recorded in autotomy crabs during the present study were due to the moulting process consequences; which correlated to a previous study by Romano and Zeng (2006), where the sustained death that happened during or after moult known as “Moult Death Syndrome” (MDS) which has been previously linked with high temperatures, genetic factors (Rouse & Kartamulia 1992), and inadequate nutrition (Browser & Rosemark 1981; Gong *et al*. 2008). Moreover, crabs in low and intermediate salinity (10 ppt and 20 ppt) showed high survivability might due to proper handling of osmotic stress compared to crabs reared in 30 ppt.

According to Amin-Safwan *et al*. (2019b), at higher salinity, mud crabs focused their energy mainly on their survival, therefore affecting their growth and development. Their study reported that, when *S. olivacea* is exposed to high salinity in a certain period (60 days), this species needs to handle the high pressure of osmotic stress, and eventually affecting their appetite, nutritional taken, movement, and therefore might negatively impact on the energy requirement for the moulting purposes. However, the adaptation might occur as organism tolerances ability might be shifted depending on surrounding and survival requirements. Therefore, this study provides information regarding the optimum water salinity for moulting performances of *S. olivacea* in-captivity. The optimum salinity level was 20 ppt, followed by 30 ppt and 10 ppt, with limb autotomy recorded as one of the best techniques to induce mud crab to moult. As a recommendation, both the manipulation of salinity (20 ppt) and limb autotomy might be one of the essential methods to induce the moulting process of *S. olivacea* in-captivity, focusing on soft-shell production and fattening process. Additionally, a higher number of crab samples (including male crabs) and wider ranges of salinity treatments should be evaluated to determine a more precise salinity effect. A longer study period (moulting cycle) is suggested to obtain a clearer pattern among these parameters to fully understand the mechanism of salinity and limb autotomy in inducing moulting performances of *S. olivacea*. Moreover, evaluation on hormone levels also might add more interesting value for future investigation.

## CONCLUSION

Based on these findings, there were significant differences between moulting duration, BW increments, growth performances and feeding efficiency with salinity. However, CW increments and survival indicates no significant effect with salinity. Meanwhile, moulting differences, BW increments, survival and feeding efficiency were affected by limb autotomy. Overall, 20 ppt was the best salinity to stimulate *S. olivacea* moulting performances and development compared with the other two treatments (10 ppt and 30 ppt). Limb autotomy also indicates promising results as this technique proved to accelerate the moulting duration of *S. olivacea*. Therefore, we highly recommend a combination of salinity (20 ppt) and limb autotomy as the best method to induce moulting performances of female *S. olivacea* in-captivity.

## Figures and Tables

**Table 1:** Effects of water salinity (10 ppt, 20 ppt and 30 ppt) and limb autotomy (claws and walking leg) on culture duration (days) of *S. olivacea*. Values are presented as average ± standard deviation (*n* = 20). Different subscripts of the alphabet are significant at *p* < 0.05 according to Tukey’s Test.

Salinity treatment (ppt)	Culture duration (days)	

	Non-ablated	Ablated
10	28.00 ± 1.41 ^a^	24.05 ± 3.44 ^b^
20	27.18 ± 1.62 ^a^	23.55 ± 2.92 ^b^
30	28.88 ± 1.36 ^a^	27.28 ± 4.08 ^a^

**Table 2:** Effects of water salinity (10 ppt, 20 ppt and 30 ppt) and limb autotomy (claws and walking leg) on CW increments of *S. olivacea*. Values are presented as average ± standard deviation (*n* = 20). No significant difference was observed among treatments.

Salinity treatment (ppt)	CW increments (%)	

	Non-ablated	Ablated
10	10.87 ± 3.44	10.38 ± 3.45
20	10.18 ± 2.37	11.14 ± 4.01
30	9.93 ± 2.25	9.49 ± 4.14

**Table 3:** Effects of water salinity (10 ppt, 20 ppt and 30 ppt) and limb autotomy (claws and walking leg) on BW increments of *S. olivacea*. Values are presented as average ± standard deviation (*n* = 20). Different subscripts of the alphabet are significant at *p* < 0.05 according to Tukey’s Test.

Salinity treatment (ppt)	BW increments (%)	

	Non-ablated	Ablated
10	9.13 ± 5.52 ^a^	7.23 ± 1.01 ^b^
20	11.08 ± 2.65 ^a^	9.16 ± 1.46 ^a^
30	10.19 ± 1.59 ^a^	11.17 ± 1.17 ^c^

**Table 4:** Effects of water salinity (10 ppt, 20 ppt and 30 ppt) and limb autotomy (claws and walking leg) on SGR of *S. olivacea*. Values are presented as average ± standard deviation (*n* = 20). No significant difference was observed among treatments.

Salinity treatment (ppt)	SGR (%^-day^)	

	Non-ablated	Limb autotomy
10	6.88 ± 2.27	7.09 ± 4.40
20	8.22 ± 1.14	8.42 ± 2.70
30	7.60 ± 0.44	7.69 ± 3.54

**Table 5:** Effects of water salinity (10 ppt, 20 ppt and 30 ppt) and limb autotomy (claws and walking leg) on survival of *S. olivacea*. Values are presented as average ± standard deviation (*n* = 20). No significant difference was observed among treatments.

Salinity treatment (ppt)	Survival (%)	

	Non-ablated	Limb autotomy
10	100	95.00 ± 8.66
20	100	91.67 ± 14.43
30	100	83.33 ± 14.43

**Table 6:** Effects of water salinity (10 ppt, 20 ppt and 30 ppt) and limb autotomy (claws and walking leg) on feeding efficiency of *S. olivacea*. Values are presented as average ± standard deviation (*n* = 60). No significant difference was observed among treatments.

Salinity treatment (ppt)	Feeding efficiency (%)	

	Non-ablated	Limb autotomy
10 ppt	32.49 ± 1.92	31.44 ± 3.20
20 ppt	40.98 ± 10.38	39.28 ± 3.07
30 ppt	35.27 ± 5.00	42.52 ± 3.96
